# Improving prescribing of antihypertensive and cholesterol-lowering drugs: a method for identifying and addressing barriers to change

**DOI:** 10.1186/1472-6963-4-23

**Published:** 2004-09-03

**Authors:** Atle Fretheim, Andrew D Oxman, Signe Flottorp

**Affiliations:** 1 Informed Choice Research Department, Norwegian Health Services Research Centre, P.O. Box 7004, St. Olavs plass, Oslo, Norway

## Abstract

**Background:**

We describe a simple approach we used to identify barriers and tailor an intervention to improve pharmacological management of hypertension and hypercholesterolaemia. We also report the results of a post hoc exercise and survey we carried out to evaluate our approach for identifying barriers and tailoring interventions.

**Methods:**

We used structured reflection, searched for other relevant trials, surveyed general practitioners and talked with physicians during pilot testing of the intervention. The post hoc exercise was carried out as focus groups of international researchers in the field of quality improvement in health care. The post hoc survey was done by telephone interviews with physicians allocated to the experimental group of a randomised trial of our multifaceted intervention.

**Results:**

A wide range of barriers was identified and several interventions were suggested through structured reflection. The survey led to some adjustments. Studying other trials and pilot testing did not lead to changes in the design of the intervention. Neither the post hoc focus groups nor the post hoc survey revealed important barriers or interventions that we had not considered or included in our tailored intervention.

**Conclusions:**

A simple approach to identifying barriers to change appears to have been adequate and efficient. However, we do not know for certain what we would have gained by using more comprehensive methods and we do not know whether the resulting intervention would have been more effective if we had used other methods. The effectiveness of our multifaceted intervention is under evaluation in a randomised controlled trial.

## Background

Much research has been carried out with the aim of influencing the performance of clinicians. The results have varied [1,2]. As with any human behaviour, clinical practice is difficult to change. Some strategies that have been evaluated, like passive dissemination of clinical practice guidelines, have had little or no effect on practice [3]. Others, like educational outreach visits ("academic detailing") and multifaceted interventions, may be more effective than passive interventions [1].

The reasons why clinical practice sometimes is not consistent with current best evidence varies across clinical problems and from one clinician to another. A logical consequence of this is to tailor quality improvement strategies to address specific barriers [4]. Several trials of tailored interventions have been conducted. The methods used for identifying barriers to change have varied and there is limited evidence of the relative usefulness of different approaches. However, the choice of method for identifying barriers has implications, particularly with regards to resources, since some methods are time consuming and demand the involvement of many individuals. This represents a practical and financial constraint. On the other hand, if such approaches lead to the identification of important barriers that otherwise would have been overlooked, they may be worth the effort.

In this article we describe a simple approach we have used to identify barriers to changing professional practice. This was done as the first step in a process of developing an intervention to improve the pharmacological management of hypertension and hypercholesterolaemia [5]. The intervention focused on three specific recommendations in clinical practice guidelines for hypertension and hypercholesterolaemia [6-8] based on evidence of a gap between the recommendations and current practice in Norway:

• Contrary to recommendations, physicians seem to rarely estimate the risk of cardiovascular disease before initiating treatment [9]

• Sales of thiazides are low, despite these drugs being recommended as first-line medication [10]

• Relatively few patients reach recommended treatment goals [11,12]

We also report the results of a post hoc exercise and a survey we carried out to evaluate our approach to identifying barriers and interventions.

## Methods

We developed the intervention through a process of identifying barriers to implementation of recommendations and measures specifically addressing these barriers ("tailoring"). The methods we used were structured reflection, searching for other relevant trials targeted at improving the management of hypertension or hypercholesterolaemia, conducting a survey among general practitioners and discussion with physicians during pilot testing of the intervention.

### Structured reflection

The three authors reflected over possible barriers based in part on our own experience as physicians working in primary care in Norway. We used a worksheet to structure our reflection (see Additional file 1). The worksheet included factors that might act as barriers in the practice environment, the professional environment, and related to physicians' knowledge, skills and attitudes. One worksheet was completed for each targeted behaviour: increasing the use of cardiovascular risk assessment before initiating treatment for hypertension or hypercholesterolaemia, increasing the prescribing of thiazides for the treatment of uncomplicated hypertension, and increasing the proportion of patients on medication for hypertension and hypercholesterolaemia that reach recommended treatment goals. The worksheet was used to facilitate our group discussion of possible interventions to address the identified barriers.

Our research group had recently completed a trial of a strategy for guidelines implementation when we were planning this study [13]. In that study the multifaceted intervention consisted of several passive components. Information and materials were distributed by mail and to a large degree we relied on the physicians themselves to make an effort at changing their practice. The observed changes in practice were small. In another trial we had found that the use of active sick leave for back patients was significantly increased through a proactive intervention compared to a passive one [14]. Based on these experiences our research group decided to test an active strategy in this study. Therefore we decided to use outreach visits ("academic detailing") prior to considering specific barriers.

We considered systematic reviews of interventions to improve professional practice when we designed our strategy [1]. We searched the Cochrane Group of Effective Care and Organisation of Care  trial register for trials of interventions targeted specifically at the management of hypertension or elevated cholesterol in general practice.

### Questionnaire to physicians

We surveyed general practitioners about some of the interventions about which we were uncertain after our structured reflection. The details of the survey have been described elsewhere [9]. Briefly, 265 physicians who had participated in an earlier trial conducted by our research group [13] were asked to complete a questionnaire as part of the study-evaluation. We used that opportunity to seek answers to the following questions:

1. Do physicians assess cardiovascular risk before prescribing antihypertensive or cholesterol-lowering drugs?

2. If not, would physicians be more likely to do so it they received a fee for this?

3. Do physicians comply with current regulations limiting the reimbursement of cholesterol-lowering drugs?

The last question was asked for two reasons. Firstly, we were considering making risk assessment a condition for reimbursement of the drugs. Secondly, the existing regulations were a possible barrier to adhering to our recommendations because they conflicted with these.

### Pilot testing

During pilot testing of the intervention at two practices, which were selected for convenience, comments from physicians relevant to possible barriers were noted. We also informally evaluated each component of the intervention.

### Post hoc focus groups and structured reflection exercise

After we had finished designing the intervention we had the opportunity of testing our method of structured reflection at a gathering of international researchers in the Research Based Education and Quality Improvement group (ReBEQI) , December 2003. Each participant was asked to complete a worksheet to identify barriers and possible interventions related to the low use of thiazides among general practitioners. They were randomly allocated to four different groups where they collaborated on completing the worksheet. They were also asked to grade the importance of each barrier or intervention as minor, moderate or major. We disregarded those rated as minor. We compared the results from the four groups with the barriers and interventions we had identified.

### Post hoc survey of physicians exposed to the intervention

While conducting the randomised trial to test the effectiveness of our multifaceted intervention we carried out telephone interviews with physicians allocated to the experimental group. They were asked if they adhered to our recommendations and, if not, why. The responses where noted down during the interviews.

## Results

### Barriers and interventions

**Figure 1:**
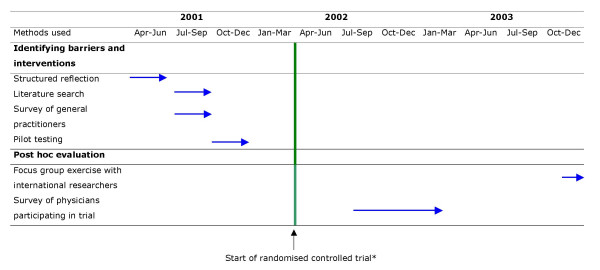
Flow chart indicating time frame for methods used to identify barriers and interventions. * The trial period ended in December 2003, and data collection and analysis will be completed in September 2004.

Figure 1 illustrates the timeframe for the methods used to identify barriers and interventions. Tables 1, 2, 3 give an overview of the barriers and interventions that we identified for each clinical problem.

Many of the barriers were related to a lack of knowledge and could be addressed through educational interventions. The use of educational outreach visits was logical since we had planned to use an active intervention, based on our previous experience, and since this type of intervention has consistently lead to improved professional behaviour in randomised trials [1]. Similarly, based on previous experience and the capabilities of the software we hade developed [13], we planned on using an electronic risk calculator, electronic prescriptions, patient information materials, and computerised reminders.

The search (July 2001) of the EPOC trial register for randomised trials with the word "hypertension" in any field yielded 58 references. Most were excluded after reading the abstracts, leaving eight, for which the full text was reviewed [15-22]. This did not lead to any changes in our intervention strategy. A search for randomised trials with the word "cholesterol" yielded 13 references. The full text was reviewed for only one of these [23]. This also provided little further guidance for designing our intervention. The nine trials that we reviewed are summarised in table 4.

The survey results did not indicate that a fee for estimating cardiovascular risk before initiating drug therapy would affect practice [9]. The survey results also indicated that physicians are largely not affected by conditions for drug reimbursement [9]. Moreover, there were no mechanisms in place to enforce such regulations.

We did not identify additional barriers during pilot testing of the intervention with five physicians in two practices, but several of those already identified were confirmed, particularly barriers to prescribing thiazides.

Based on our findings and an assessment of the feasibility and evidence of effectiveness for various interventions, we designed a multifaceted intervention. The elements of the intervention are described in table 5.

We also considered a number of interventions that we excluded. For example:

• We considered placing computers in waiting rooms so that patients could assess their cardiovascular risk before seeing the physician, but concluded this would be costly and difficult to implement.

• We considered providing pre-printed prescriptions, but found this would not to be relevant because most physicians use computerised systems for prescribing.

• We considered exposing conflicts of interest among clinical specialists who advocated using other first line drugs than thiazides, but elected not to do so.

• We considered exposing techniques used in pharmaceutical advertisements, such as using relative risk reductions rather than absolute risk reductions [24], but concluded this would have at best a limited impact.

### Post hoc focus groups and structured reflection exercise

Nineteen researchers were divided into four groups. All groups considered advocacy by drug companies to be a major barrier to change. Routines or habits were also included as an important barrier by all the groups, as well as lack of knowledge concerning the effectiveness of thiazides, their favourable adverse effects profile, and their low cost. All the groups also mentioned competing guidelines or diverging opinions as part of the problem. Three of the groups considered local or national opinion leaders as potential barriers to change. Patients' expectations or perceived expectations were also mentioned by three of the groups.

The interventions recommended by the groups to address the identified barriers are presented in table 6. All the groups suggested the use of computerised reminders to address physicians' lack of knowledge or their habits and routines. All the groups also suggested some form of interactive education, mainly as a counter force to promotional activities by the pharmaceutical industry, and patient information was suggested by three of the groups. Two suggested training physicians to address patient expectations. Two groups suggested developing clinical guidelines and two suggested audit and feedback, but one group considered this to be of minor importance.

### Post hoc survey of physicians exposed to the intervention

Among the 195 physicians exposed to the intervention, 149 (76%) were contacted during the trial period and agreed to answer our questions. No major additional barriers were identified. However, some physicians questioned whether adhering to the recommendations would represent a good use of resources, specifically the recommended treatment goals.

## Discussion

Addressing barriers to change with tailored interventions makes sense and there is some empirical support for this [1]. It is unclear, however, what methods are the most useful for identifying barriers and interventions.

Several qualitative methods can be used to identify barriers, such as interviews, focus groups and observation. These methods may be valuable, but they are relatively labour-intensive. We used a simpler approach to identifying barriers to change. Would the use of other methods have provided us with important additional information? Pilot testing and discussions with five physicians in two practices and interviews with 140 participating physicians did not indicate additional barriers. The post-hoc focus groups with international experts did not add much with regards to barriers and interventions. Several of these groups included "routines and habits" as a potential barrier, which was not explicitly mentioned among the barriers identified by the investigators. However, all interventions that were mentioned by more than one of the groups in the post-hoc focus group exercise were included in our multifaceted intervention. Our use of computerised reminders was based on the assumption that this would help to establish new routines, although we did not record routines and habits as a barrier when we developed the intervention.

There are inherent weaknesses in our approach. One is that the investigators undertaking the structured reflection were few and we were prejudiced by our own experiences. The lack of patient involvement is another limitation, which possibly lead to an under-emphasis of patient-mediated interventions. A weakness with the group of international researchers who participated in the post-hoc focus groups is their lack of familiarity with the Norwegian context.

A number of trials of tailored interventions have been conducted. The methods used to identify barriers to change have varied. Some investigators have simply hosted a meeting [25,26], others have used questionnaires [23], conducted focus-groups [27-30] or interviews [31-33], or both [34]. Others have used a combination of several qualitative methods [35-37]. Some investigators have used identification of barriers as an intervention in itself [19,38,39]. The methods that were used have been poorly described in most of these studies.

## Conclusions

Our simple approach to identifying barriers to improving practice appears to have been effective in identifying all of the important barriers, and it was efficient. However, we do not know for certain what barriers other methods would have identified or whether the intervention could have been more effective, if we had used other methods. Further work to address these questions is planned, including direct comparison of alternative methods and evaluations of theory-based approaches .

The effectiveness of our multifaceted intervention is under evaluation in a randomised controlled trial.

## Competing interests

None declared.

## Author contributions

All the authors participated in the process of structured reflection and in conducting the survey of physicians. AF was responsible for reviewing the results from previous research and pilot testing of the intervention. AF drafted the article while SF and ADO contributed to critical revisions of the manuscript. All authors read and approved the final manuscript.

## Pre-publication history

The pre-publication history for this paper can be accessed here:



## Figures and Tables

**Table 1:** Barriers to carrying out cardiovascular risk assessment, and possible interventions to address these

**Possible barriers**	**Possible interventions**
Time-consuming procedure for the physician	- Easy-to-use tools (e.g. risk calculator for computer) - The patients or an assistant could carry out the risk assessment - Offer economic compensation
Physician has no risk assessment tool at hand	- Provide risk assessment tool
The patients are focused on single risk-factors, not the global picture	- Patient-information
Physicians are not used to risk estimation, not educated to do this	- Educational outreach visit - Training
Lack of knowledge among physicians of the relevance of global risk assessment	- Information/education (outreach visit)
Physicians have more trust in their own clinical judgement than tables or charts	- Education (e.g. case discussions during outreach visit)
Differences in opinion among physicians on the importance of treatment of hypertension and hypercholesterolaemia	- Use opinion-leaders and convince clinicians of the high degree of consensus in clinical guidelines
May be uncomfortable for physicians to discuss risk-factors with patients	- Patient-information - Offer strategies on how to communicate such information with patients

**Table 2:** Barriers to prescribing thiazides for the treatment of hypertension, and possible interventions to address these

**Possible barriers**	**Possible interventions**
Physicians are neither familiar with the relevant brand-names nor to the use and follow-up of these drugs	- Pre-printed prescriptions, also in electronic format - Patient information - Support for the clinical follow-up
Few other clinicians use these drugs	- Patient information - Active promotion of thiazides (through educational outreach visits) - Point out the consensus among guidelines that thiazides are a first-line drug
Specialists may be prescribing other drugs	- Identify opinion leaders that advocate the use of thiazides - Look into possible conflicts of interest
Advocacy by pharmaceutical companies	- Point attention to the importance of clinically relevant endpoints when studies are quoted (during educational outreach visits) - Review advertisements to identify the main lines of reasoning that are being used
Physicians are worried about possible side-effects and lack of anti-hypertensive effect.	- Educational outreach visits
Thiazides considered old-fashioned	- Argue that these drugs have been thoroughly tested over many years (during educational outreach visits)

**Table 3:** Barriers to reaching recommended treatment goals and possible interventions to address them

**Possible barriers**	**Possible interventions**
Physicians are not accountable to anyone	- Feed-back on to what extent treatment goals are reached among his/her pool of patients (audit)
Physicians are unsure of what treatment goal to use	- Give clear treatment goals (during outreach visit) - Point at the relatively high degree of consensus among guidelines (during outreach visit)
Reluctance and unclear strategy among physicians on how to deal with insufficient treatment	- Support for decision-making if goal is not reached
Physicians may be underestimating the consequence of under-treatment	- Educational outreach visit

**Table 4:** Studies targeting the management of hypertension and/or hypercholesterolaemia

**Study**	**Objective**	**Intervention**	**Comment**
Bass 1986 [15]	Improve the detection and management of hypertension	Medical assistant oversaw screening, attended to education, compliance and follow-up	We did not consider this to be a feasible intervention in our setting
Aucott 1996 [19]	Implement guidelines for cost-effective management of hypertension on medication use and cost, blood pressure control, and other resource use	Intensive guideline-based education and supervision (identification of clinical champion, faculty education and development, assignment of PharmD, clinic-based education and precepting of clinicians, monthly feedback to practice)	Most elements of this multifaceted intervention were already included in our own. The trial was conducted in a general internal medicine teaching clinic, which limits the relevance to our primary care setting
Rossi 1997 [16]	Alter prescribing habits for the treatment of hypertension	Guideline reminders placed in the charts of patients	Computerised reminders were already included as part of our multifaceted intervention
Goldberg 1998 [17]	Increase compliance with national guidelines for the primary care of hypertension (and depression)	Academic detailing with or without continuous quality improvement (CQI) teams	Academic detailing (outreach visit) was already included as part of our multifaceted intervention. The study-findings did not support the use of CQI teams
Maclure 1998 [18]	Increase understanding of the way in which dissemination of evidence changes medical practice	Media stories, national warning letter, teleconference, small group workshops, and newsletters	Our outreach visits were planned as interactive sessions, thus serving the same purpose as small group workshops or teleconferences. We did not believe that passive distribution of material would be useful
Hetlevik 1998 [21]	Implement clinical guidelines in the treatment of hypertension	Computer based clinical decision support system, mailed feedback of current practice, invitation to seminar at conference	Most interventions were already included in our multifaceted intervention. We did not believe that inviting to conference-seminar would be useful
van der Weijden 1999 [23]	Assess the feasibility and evaluation needs of a cholesterol guideline	Group education, desktop supportive materials, feedback on performance, and face-to-face instruction on location	Most interventions were already included in our multifaceted intervention
Montgomery 2000 [20]	Have an effect on absolute cardiovascular risk, blood pressure, and prescribing of cardiovascular drugs	Computer based clinical decision support system plus cardiovascular risk chart; or cardiovascular risk chart alone	Both interventions were already included in our multifaceted intervention
Demakis 2000 [22]	Improve resident physician compliance with standards of ambulatory care (including hypertension)	Computerised reminder system	Intervention was already included in our multifaceted intervention

**Table 5:** The final multifaceted intervention

**Educational outreach visit**
- Presentation focusing on three main messages:
1. Relevance of risk estimation and how to do it, including strategies on how to communicate information about risk to patients.
2. Information on evidence in support of effect and the unjustified fear of adverse effects regarding thiazides, pointing at the consensus that exists among guidelines. Attention also directed to the importance of clinically relevant endpoints when studies are quoted.
3. Clear recommendations justified by referring to high degree of consensus among guidelines.
- Guidelines handed out, directing attention to the authors (opinion leaders)

**Audit & feed-back at outreach visit**

- To what extent treatment goals are achieved.
- Drug-choice profile on anti hypertensives
- Level of risk among patients on treatment, compared to a sample (men 40–65 years) not on treatment

**Computerised reminders**

- Risk assessment
- First-choice antihypertensive drugs
- Treatment goals

**Risk assessment tools as charts and in electronic format**

**Patient-information material**

- The relationship between single risk factors and global risk
- Thiazides and beta-blockers.
- Treatment goals

**Table 6:** Interventions to address identified barriers (main results from post-hoc focus group and structured reflection exercise)

**Barrier: Marketing activities by pharmaceutical industry**			
	Importance of barrier*	**Interventions**	Importance of interventions*

Group 1	3	- Competing approaches (educational materials; interactive educational workshops)	2
Group 2	3	- Outreach visits	3
Group 3	3	- Small group peer comparison	1–2
Group 4	3	- Continuing education system	Not graded

**Barrier: Routines and habits**			

	Importance of barrier*	**Interventions**	Importance of interventions*

Group 1	3	- Computerised reminders	3
		- Audit and feedback	2
		- Intention plus/trial of behaviour	2
Group 2	2	- Reminder	3
		- Direct mail	3
Group 3	2/3	- Audit and feed back with peer comparison	1
Group 4	Not graded	- Computerised reminders	Not graded

**Barrier: Lack of knowledge**			

	Importance of barrier*	**Interventions**	Importance of interventions*

Group 1	2	- Educational material/guidelines	2
		- Interactive educational meetings	2
Group 2	2	- Information to patients	2
		- Local quality circles	2
Group 3	Not graded	- Financial incentives	3
		- Reminders/Computerised Decisions Support Systems	2
Group 4	Not graded	- Continuing medical education	Not graded
		- Computerised reminders	

**Barrier: Opinion leaders (or specialists) or competing guidelines**			

	Importance of barrier*	**Interventions**	Importance of interventions*

Group 1	3	- Develop national guidelines	2
Group 2	3	- Use opinion leaders	3
Group 3	2	- Guidelines shared by primary and specialist physicians	1–2
Group 4	Not graded	- Not explicitly addressed	-

**Barrier: Patient expectations**			

	Importance of barrier*	**Interventions**	Importance of interventions*

Group 1	2	- Patient materials	3
		- Educational meetings for general practitioners	2
Group 2	Not mentioned	- None	-
Group 3	Not graded	- Skills programme training	2
Group 4	Not graded	- Information leaflet to patients about options	Not graded

*1 = minor, 2 = moderate, 3 = major

## References

[B1] Grimshaw JM, Shirran L, Thomas R, Mowatt G, Fraser C, Bero L, Grilli R, Harvey E, Oxman A, O'Brien MA (2001). Changing provider behavior: an overview of systematic reviews of interventions. Med Care.

[B2] Oxman AD, Thomson MA, Davis DA, Haynes RB (1995). No magic bullets: a systematic review of 102 trials of interventions to improve professional practice. CMAJ.

[B3] Freemantle N, Harvey EL, Wolf F, Grimshaw JM, Grilli R, Bero LA (2001). Printed educational materials: effects on professional practice and health care outcomes. The Cochrane Library.

[B4] Oxman AD, Flottorp S (2001). An overview of strategies to promote implementation of evidence base health care. Evidence Based Practice.

[B5] Fretheim A, Oxman A, Treweek S, Bjørndal A (2003). Rational Prescribing in Primary Care (RaPP-trial). A randomised trial of a tailored intervention to improve prescribing of antihypertensive and cholesterol-lowering drugs in general practice [ISRCTN48751230]. BMC Health Services Research.

[B6] Fretheim A, Bjørndal A, Oxman AD, Dyrdal A, Golding M, Ose L, Reikvam A, Teisberg P (2002). Retningslinjer for medikamentell primærforebygging av hjerte- og karsykdom – hvem bør behandles? [Guidelines for pharmacological primary prevention of cardiovascular disease: who should be treated?]. Tidsskr Nor Laegeforen.

[B7] Fretheim A, Bjørndal A, Oxman AD, Dyrdal A, Golding M, Ose L, Reikvam A, Teisberg P (2002). Hvilke blodtrykkssenkende legemidler bør brukes for primærforebygging av hjerte- og karsykdommer? [Which antihypertensive drugs should be used in the primary prevention of cardiovascular disease?]. Tidsskr Nor Laegeforen.

[B8] Fretheim A, Bjørndal A, Oxman AD, Dyrdal A, Golding M, Ose L, Reikvam A, Teisberg P (2002). Hvilke kolesterolsenkende legemidler bør brukes for primærforebygging av hjerte- og karsykdommer? [Which cholesterol-lowering drugs should be used in the primary prevention of cardiovascular disease?]. Tidsskr Nor Laegeforen.

[B9] Fretheim A, Havelsrud K, Flottorp S, Oxman AD (2003). Påvirker takster og refusjonsregler praksis? [Do fees for service and regulations for reimbursement influence practice?]. Tidsskr Nor Laegeforen.

[B10] Norwegian Institute of Public Health (2002). Drug Consumption in Norway 1997 – 2001 Oslo.

[B11] Svilaas A, Risberg K, Thoresen M, Ose L (2000). Lipid treatment goals achieved in patients treated with statin drugs in Norwegian general practice. Am J Cardiol.

[B12] Westheim A, Klemetsrud T, Tretli S, Stokke HP, Olsen H (2001). Blood pressure levels in treated hypertensive patients in general practice in Norway. Blood Press.

[B13] Flottorp S, Oxman AD, Havelsrud K, Treweek S, Herrin J (2002). A cluster randomised trial of tailored interventions to improve the management of urinary tract infections and sore throat. BMJ.

[B14] Scheel IB, Hagen KB, Herrin J, Oxman AD (2002). A randomized controlled trial of two strategies to implement active sick leave for patients with low back pain. Spine.

[B15] Bass MJ, McWhinney IR, Donner A (1986). Do family physicians need medical assistants to detect and manage hypertension?. CMAJ.

[B16] Rossi RA, Every NR (1997). A computerized intervention to decrease the use of calcium channel blockers in hypertension. J Gen Intern Med.

[B17] Goldberg HI, Wagner EH, Fihn SD, Martin DP, Horowitz CR, Christensen DB, Cheadle AD, Diehr P, Simon G (1998). A randomized controlled trial of CQI teams and academic detailing: can they alter compliance with guidelines?. Jt Comm J Qual Improv.

[B18] Maclure M, Dormuth C, Naumann T, McCormack J, Rangno R, Whiteside C, Wright JM (1998). Influences of educational interventions and adverse news about calcium-channel blockers on first-line prescribing of antihypertensive drugs to elderly people in British Columbia. Lancet.

[B19] Aucott JN, Pelecanos E, Dombrowski R, Fuehrer SM, Laich J, Aron DC (1996). Implementation of local guidelines for cost-effective management of hypertension. A trial of the firm system. J Gen Intern Med.

[B20] Montgomery AA, Fahey T, Peters TJ, MacIntosh C, Sharp DJ (2000). Evaluation of computer based clinical decision support system and risk chart for management of hypertension in primary care: randomised controlled trial. BMJ.

[B21] Hetlevik I, Holmen J, Kruger O, Kristensen P, Iversen H (1998). Implementing clinical guidelines in the treatment of hypertension in general practice. Blood Press.

[B22] Demakis JG, Beauchamp C, Cull WL, Denwood R, Eisen SA, Lofgren R, Nichol K, Woolliscroft J, Henderson WG (2000). Improving residents' compliance with standards of ambulatory care: results from the VA Cooperative Study on Computerized Reminders. JAMA.

[B23] van der Weijden T, Grol RP, Knottnerus JA (1999). Feasibility of a national cholesterol guideline in daily practice. A randomized controlled trial in 20 general practices. Int J Qual Health Care.

[B24] Skolbekken JA (1998). Communicating the risk reduction achieved by cholesterol reducing drugs. BMJ.

[B25] Soumerai SB, McLaughlin TJ, Gurwitz JH, Guadagnoli E, Hauptman PJ, Borbas C, Morris N, McLaughlin B, Gao X, Willison DJ, Asinger R, Gobel F (1998). Effect of local medical opinion leaders on quality of care for acute myocardial infarction: a randomized controlled trial. JAMA.

[B26] (1995). A controlled trial to improve care for seriously ill hospitalized patients. The study to understand prognoses and preferences for outcomes and risks of treatments (SUPPORT). The SUPPORT Principal Investigators. JAMA.

[B27] Flottorp S, Oxman AD (2003). Tailoring interventions to improve the management of urinary tract infections and sore throat: a pragmatic study using qualitative methods. BMC Health Services Research.

[B28] Evans D, Mellins R, Lobach K, Ramos-Bonoan C, Pinkett-Heller M, Wiesemann S, Klein I, Donahue C, Burke D, Levison M, Levin B, Zimmerman B, Clark N (1997). Improving care for minority children with asthma: professional education in public health clinics. Pediatrics.

[B29] Hux JE, Melady MP, DeBoer D (1999). Confidential prescriber feedback and education to improve antibiotic use in primary care: a controlled trial. CMAJ.

[B30] Santoso B, Suryawati S, Prawaitasari JE (1996). Small group intervention vs formal seminar for improving appropriate drug use. Soc Sci Med.

[B31] Avorn J, Soumerai SB, Everitt DE, Ross-Degnan D, Beers MH, Sherman D, Salem-Schatz SR, Fields D (1992). A randomized trial of a program to reduce the use of psychoactive drugs in nursing homes. N Engl J Med.

[B32] Majumdar SR, Soumerai SB, Lee M, Ross-Degnan D (2001). Designing an intervention to improve the management of Helicobacter pylori infection. Jt Comm J Qual Improv.

[B33] Avorn J, Soumerai SB (1983). Improving drug-therapy decisions through educational outreach. A randomized controlled trial of academically based "detailing". N Engl J Med.

[B34] Leviton LC, Baker S, Hassol A, Goldenberg RL (1995). An exploration of opinion and practice patterns affecting low use of antenatal corticosteroids. Am J Obstet Gynecol.

[B35] Ross-Degnan D, Soumerai SB, Goel PK, Bates J, Makhulo J, Dondi N, Sutoto, Adi D, Ferraz-Tabor L, Hogan R (1996). The impact of face-to-face educational outreach on diarrhoea treatment in pharmacies. Health Policy Plan.

[B36] Forsetlund L, Bjorndal A (2002). Identifying barriers to the use of research faced by public health physicians in Norway and developing an intervention to reduce them. J Health Serv Res Policy.

[B37] Scheel IB, Hagen KB, Oxman AD (2002). Active sick leave for patients with back pain: all the players onside, but still no action. Spine.

[B38] Baker R, Reddish S, Robertson N, Hearnshaw H, Jones B (2001). Randomised controlled trial of tailored strategies to implement guidelines for the management of patients with depression in general practice. Br J Gen Pract.

[B39] Cranney M, Barton S, Walley T (1999). Addressing barriers to change: an RCT of practice-based education to improve the management of hypertension in the elderly. Br J Gen Pract.

